# Biocontrol of *Aspergillus flavus* in Ensiled Sorghum by Water Kefir Microorganisms

**DOI:** 10.3390/microorganisms7080253

**Published:** 2019-08-10

**Authors:** Mariana Gonda, Gabriela Garmendia, Caterina Rufo, Ángela León Peláez, Michael Wisniewski, Samir Droby, Silvana Vero

**Affiliations:** 1 Área Microbiología, Departamento de Biociencias, Facultad de Química, Universidad de la República, Gral Flores 2124, Montevideo 11800, Uruguay; 2 Instituto Polo Tecnológico, Facultad de Química, Universidad de la República, By Pass Ruta 8 s/n, Pando, 8 Canelones 90000, Uruguay; 3 Cátedra de Microbiología, Facultad de Ciencias Exactas, Universidad Nacional de La Plata, Calle 47 y 115, La Plata 1900, Argentina; 4 Appalachian Fruit Research Station, Agricultural Research Service, United States Department of Agriculture, Wiltshire Road, Kearneysville, WV 25443, USA; 5 Agricultural Research Organization (ARO), Department of Postharvest Science, The Volcani Center, Rishon LeZion 7505101, Israel

**Keywords:** water kefir, high moisture sorghum silage, *Aspergillus flavus*, high-throughput sequencing

## Abstract

The capacity of microorganisms from water kefir (WK) to control *Aspergillus flavus* growth during the aerobic phase of ensiled sorghum grains was determined. Sorghum inoculated with *A. flavus* was treated with filter-sterilized and non-sterilized water kefir, ensiled, and incubated 7 days at 25 °C. *A. flavus* growth was quantified by qPCR after incubation. Mold growth was inhibited in the presence of water kefir while no inhibition was observed when filter-sterilized water kefir was applied, demonstrating the relevant role of the microorganisms in the kefir water in the biocontrol process. Fungal and bacterial diversity in treated sorghum mini-silos was analyzed by high-throughput sequencing. Firmicutes was the predominant bacterial phyla and *Lactobacillus* represented the most abundant genus, while Ascomycota was the predominant fungal phyla with *Saccharomyces* and *Pichia* as the major genera. Bacterial and yeast counts before and after incubation indicated that the microbial community obtained from WK was able to grow in the sorghum mini-silos in the presence of *A. flavus*. Results of the present work indicate that the use of a mixed inoculum of microorganisms present in WK may represent an alternative management practice to avoid the growth of *A. flavus* in ensiled sorghum grains and the concomitant contamination with aflatoxins.

## 1. Introduction

Silage plays a significant role as a source of animal feed, especially in areas where the demand for feed is year-round. It is an essential component of the diet of ruminants when fresh dietary crops are unavailable, as in winter [1]. High-moisture grain silage in Uruguay is an important food resource for livestock and dairy animals. It represents a higher quality energy source than dry grain, is economically cheaper, and its production is less dependent on weather conditions [2]. The process of producing silage involves several stages that require a total of 30–40 days, depending on the ensiling material [3]. The silage process can be divided into four phases; an initial aerobic phase immediately after harvest; a fermentation phase; a stable storage phase, and lastly, a feed-out phase when the silo feed face is open and the material is exposed to air [4]. The exposure of silage to oxygen during storage and feed-out phases has a negative impact on silage quality as it allows fungi to proliferate. Fungal growth in silage results in a loss of nutrients and dry matter, and reduced palatability resulting in a reduction in silage consumption, both of which cause a loss in animal performance [5]. Some spoilage molds can also produce mycotoxins that can cause the animals to become intoxicated. Genera associated with silage deterioration are *Aspergillus*, *Penicillium*, and *Fusarium* [6]. Previous work of García y Santos (2012) [7] performed in Uruguay, indicated that *Aspergillus flavus* was the main contaminant species in sorghum silage. These results are in agreement with Keller et al. (2012) [8], who identified *Aspergillus flavus* as the main spoiler of sorghum silage in the south of Brazil. *A. flavus* produces aflatoxins that are carcinogenic and mutagenic mycotoxins [9]. Consumption of aflatoxin-contaminated feed is associated with reduced animal performance. 

Different strategies, including biological control, have been used to prevent fungal growth in silages [6]. Bacteria and yeast have been described as good biocontrol agents to prevent mold growth in high moisture grain silage. *Lactobacillus* spp. are commonly used as a silage inoculant due their ability to produce lactic acid, which causes a quick decrease in the pH of the silage substrate, limiting the growth of many microbial contaminants. Lactic acid bacteria also compete with spoilage microorganisms and minimize gas losses and proteolysis [10]. They have antibacterial and antifungal activity and the capacity to adsorb mycotoxins [11]. The use of yeast as biocontrol agents for the control of mycotoxigenic molds in ensiled grains has also been studied and yeast has been shown to inhibit fungal growth and decrease the risk of mycotoxin contamination in silage [12,13]. Yeast also improve the nutritional value of silage by increasing vitamin and protein content. [14,15]. *Saccharomyces* spp. are used as a direct-fed microbial (DFM), to improve feeding efficiency, decrease ruminal acidosis, and mitigate methane emissions [16]. 

Schnürer and Jonsson (2011) [17] suggested that an excellent starter culture for grain silage should include a combination of microorganisms, in particular yeast and lactic acid bacteria. Yeasts may control mold growth, especially in the first step of the ensiling process, by contributing to oxygen depletion, then when oxygen is low or absent, lactic acid bacteria can inhibit mold growth by decreasing pH through the secretion of organic acids and by the production of antimicrobial compounds. The use of natural and synthetic microbial consortia represents an emerging field in the biocontrol of plant pathogens or microbial spoilage of food and feed [18,19,20]. Microbial consortia often have the ability to complete tasks that could not be accomplished by a single strain. Therefore, their use can result in a more efficient and robust process that is less affected by environmental stress [21].

The object of the present study was to examine the potential of microorganisms from water kefir grains as potential biocontrol agents. Kefir grains consist of a complex association of bacteria and yeast, bound within a dextran matrix. This association involves many different yeast species, lactic acid bacteria, and some species of acetic acid bacteria [22]. Water kefir is produced by the fermentation of a sugary solution by microorganisms present in kefir grains, referred to as a starter culture [22,23]. Kefir is recognized as an excellent source of probiotics with potential health benefits [24,25,26]. Antioxidant, anti-inflammatory, antifungal, and antibacterial activity are among the many health benefits that have been demonstrated [19,27,28]. A variety of pharmaceutical attributes have been associated with specific strains of yeast and bacteria isolated from kefir [29,30]. This suggests that water kefir may represent an excellent source of single strains or microbial consortia to be used in managing the silage process and preventing the establishment of microorganisms that diminish the quality and safety of silage. The specific aim of the present work was to study the ability of the microorganisms present in water kefir to act as biocontrol agents against *Aspergillus flavus* during the aerobic phase of the silage process in sorghum silages. 

## 2. Results

### 2.1. Quantification of A. flavus DNA by qPCR

**Figure 1:**
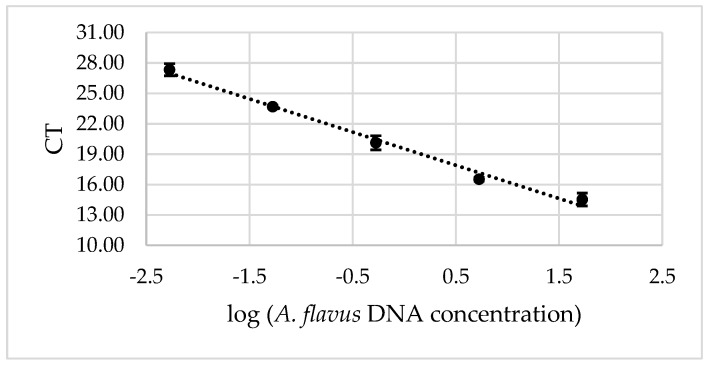
Standard curve for the real time PCR assay of *A. flavus* DNA. Bars represent confidence intervals (α = 0.05).

The standard curve generated revealed a strong linear relationship (R^2^ = 0.99) between the logarithm of *A. flavus* DNA concentration (ng/µL) in the reaction tube and the corresponding CT values (Figure 1). Linearity was observed over the range between 53 ng to 5.3 × 10^−3^ ng of DNA per reaction, which constituted the dynamic range of the method. Based on this calculation and the treatment of samples prior to performing qPCR, the quantification limit of the method expressed in terms of sample weight was 3.2 ng DNA/g. Efficiency calculated from the slope of the curve was 102%, indicating that the qPCR assay was highly efficient. 

### 2.2. A. flavus Growth in Mini-Silos

**Figure 2:**
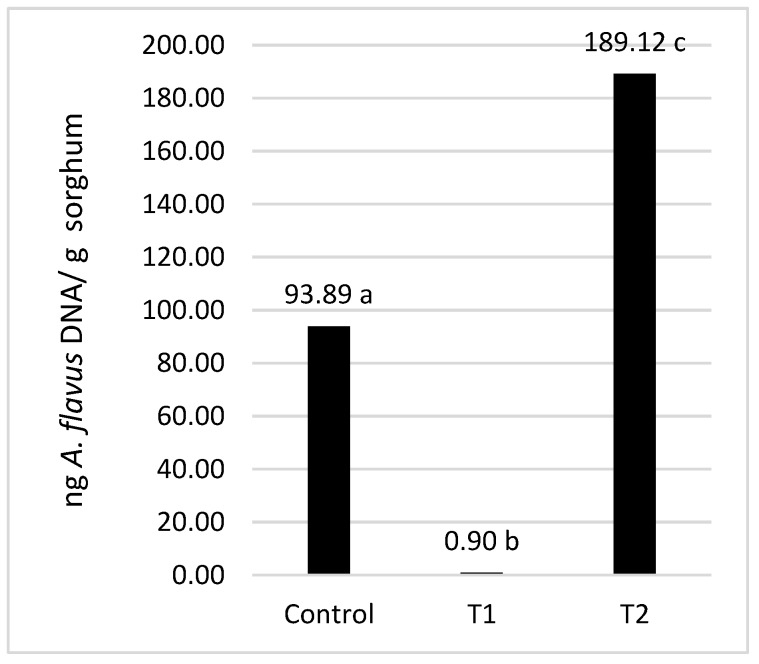
Nanograms (ng) of *A. flavus* DNA per sorghum weight obtained from the control (sterile water), T1 (water kefir), and T2 (filter-sterilized water kefir) treatments. Values labeled with different letters are significantly different, as calculated by a LSD Fisher Test α = 0.05.

**Figure 3:**
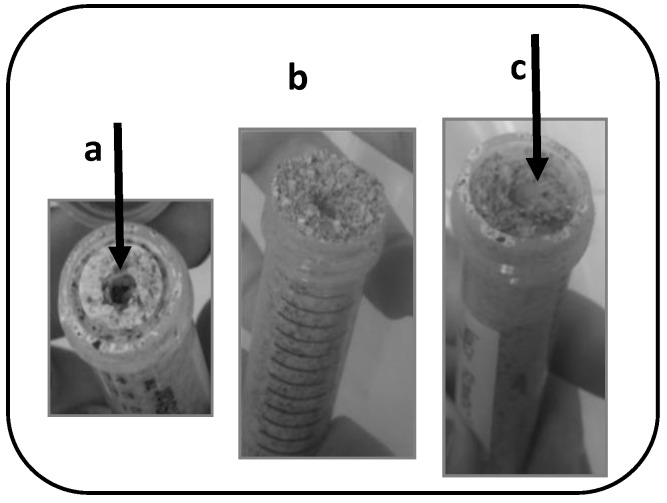
Experimental high moisture sorghum grain mini-silos opened after 7 days of incubation at 25°C. (**a**) Control (sterile water); (**b**) T1 (water kefir consortium); (**c**) T2 (filter-sterilized water kefir). Arrows indicate *A. flavus* growth.

*A. flavus* PJA readily grew in both the control and T2 mini-silos under the assayed conditions. In control mini-silos, which were prepared with sorghum and sterile water, *A. flavus* DNA concentration after incubation was 93.9 ng of DNA/g sorghum while, in T2 mini-silos, a significantly higher concentration (189.1 ng of DNA/g sorghum) was detected (Figure 2). In both treatments, fungal growth was easily visible when the mini-silos were opened (Figure 3). 

Visible symptoms of *A. flavus* growth were not observed in T1 mini-silos (Figure 2 and Figure 3). The estimated DNA concentration of the mold as quantified by qPCR was also lower than the quantification limit of the method established for 3.2 ng of DNA/g sorghum. An estimated value of 0.9 ng of DNA/g sorghum was obtained by extrapolation. That concentration was significantly lower (α = 0.05) than was obtained in the control and T2 mini-silos (Figure 2), indicating that the microorganisms present in the WK mixture played a significant role in inhibiting the growth of *A. flavus*.

### 2.3. Microbiological Analysis of T1 Mini-Silos

**Figure 4:**
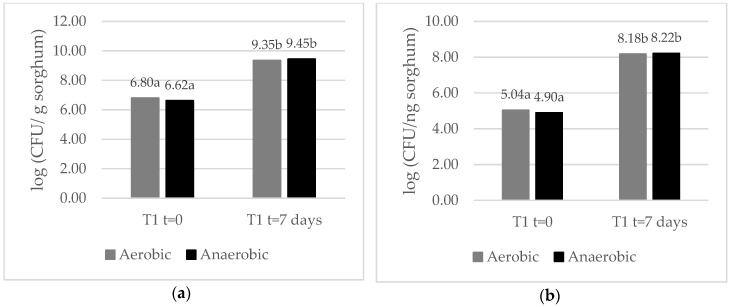
(**a**) Bacterial plate count per sorghum weight in aerobic and anaerobic condition. (**b**) Yeast plate count per sorghum weight in aerobic and anaerobic conditions. Values labeled with different letters are significantly different, as calculated by a LSD Fisher Test α = 0.05.

As illustrated in Figure 4a,b, the concentration of lactic and acetic acid bacteria and yeast increased significantly in T1 mini-silos incubated at 25 °C from day 0 to day 7 (Figure 4a,b). No significant differences were observed, however, in bacterial and yeast counts obtained from mini-silos incubated under aerobic or anaerobic conditions (Figure 4a,b).

Five bacterial colonies that differed in morphology were isolated from T1 mini-silos before (1AB, 2AB, 3AB, 4AB, 5AB) and after (1DB, 2DB, 3DB, 4DB, 5DB) the seven-day incubation period. Six of the isolates (1AB, 3AB, 5AB, 1DB, 3DB, 5DB) were Gram-positive rods with slight differences in their microscopic morphology. The other four isolates were Gram-negative rods. In the case of yeasts, two isolates with different colony types were obtained before (1AL, 2AL) and after (1DL, 2DL) incubation. The ratio of the abundance (number of colonies) of these isolates was 1:3 before and after incubation

**Figure 5:**
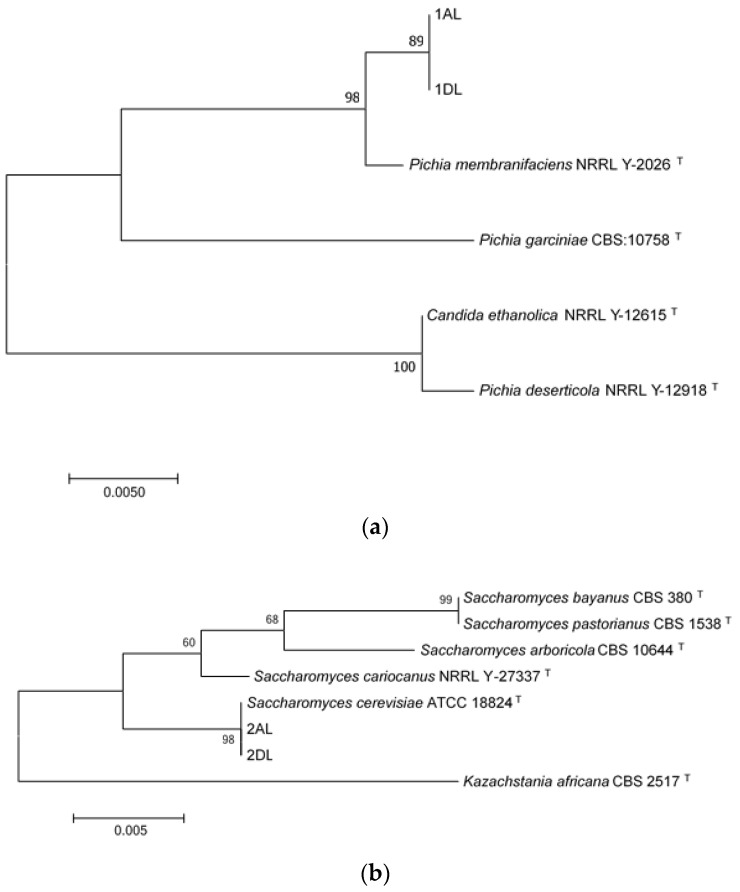
Phylogenetic trees based on D1/D2 sequences from (**a**) *Pichia membranifaciens*. (**b**) *Saccharomyces cerevisiae* isolates. Trees were constructed using the neighbor-joining method. Bootstrap values (1000 tree interactions) are indicated at the nodes. ^T-^ means type strain.

The yeast isolates were identified based on the D1/D2 consensus sequence. The D1/D2 sequences from 1AL and 1AD isolates had a 99.58% and 99.61% homology, respectively, to *Pichia membranifaciens*, type strain NRRL Y-2026 in GenBank (EU057561.1). The second most-related species was *Pichia garciniae* with a 96% similarity. D1/D2 sequences of isolates 2AL and 2DL exhibited a 100% homology to *Saccharomyces cerevisiae* ATCC 18824 type strain in GenBank (KC 881066.1). The second most related specie to 2AL and 2DL was *Saccharomyces cariocanus* with a 99% similarity. Phylogenetic trees derived from an analysis of the 26S rDNA domain D1/D2 of each group of strains are presented in Figure 5. Isolates 1AL and 1DL formed a separate cluster with *Pichia membranifaciens* type strain NRRL Y-2026, while isolates 1AL and 1DL formed a separate cluster with *Saccharomyces cerevisiae* type strain ATCC 18824, confirming in each case their identification. 

**Figure 6:**
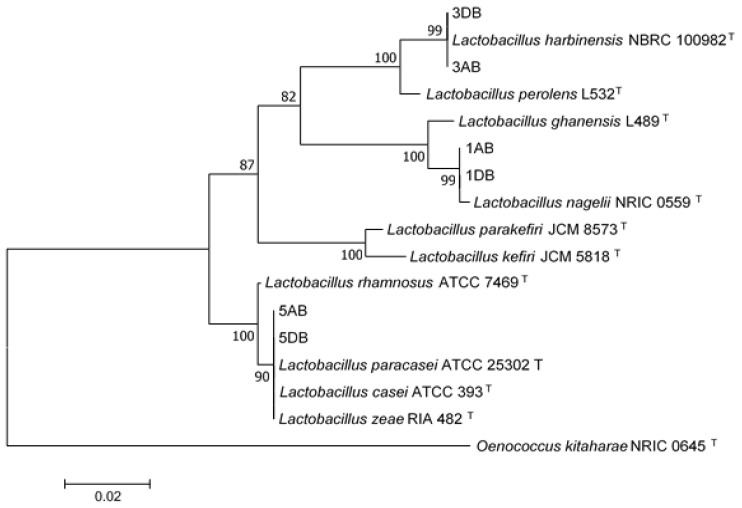
Phylogenetic tree based on the partial sequence of the 16S rRNA gene of *Lactobacillus* spp. isolates. The tree was constructed using the neighbor-joining method. Bootstrap values (1000 tree interactions) are indicated at the nodes. ^T-^ means type strain.

Based on the obtained sequences of 16S rDNA, the bacterial isolates were identified as *Acetobacter sp.* (2AB and 2DB), *Gluconobacter sp.* (4AB and 4DB), and *Lactobacillus sp.* (1AB, 1DB, 3AB, 3DB, 5AB, 5DB). A phylogenetic tree was constructed based on the 16S rRNA sequences of strains identified as *Lactobacillus sp.* and type strain sequences from related species retrieved from GenBank. Isolates 3AB and 3DB formed a separate cluster with *Lactobacillus harbinensis* type strain NBRC 100982, suggesting that those isolates may belong to this species (Figure 6). Isolates 1AB and 1DB grouped with the type strain of *Lactobacillus nagelii* NRIC 0559, and isolates 5AB and 5DB formed a cluster with species belonging to *Lactobacillus casei* group [31] (Figure 6).

### 2.4. Data Analysis of High-Throughput Amplicon Sequencing

A total of 175,936 sequences of the V4 region of the 16S rRNA gene were obtained from T1 mini-silos before (66,571) and after (109,365) incubation. In addition, 153,509 ITS2 sequence reads were obtained for the fungal community.

The number of bacterial OTUs obtained before and after the incubation of T1 mini-silos was 41 and 51, respectively, while they were 21 and 13 in case of fungi. Chao1 and Shannon indexes were calculated for each data set (Table 1). The Chao1 estimator of richness for bacteria was 42.7 before and 52.5 after incubation, while for fungi they were 21.2 and 16.0, respectively. Shannon diversity indexes were practically the same before and after incubation for both bacterial and fungal communities. Based on the obtained values, it appears that the fungal community had a lower level of diversity than the bacterial community. 

**Figure 7:**
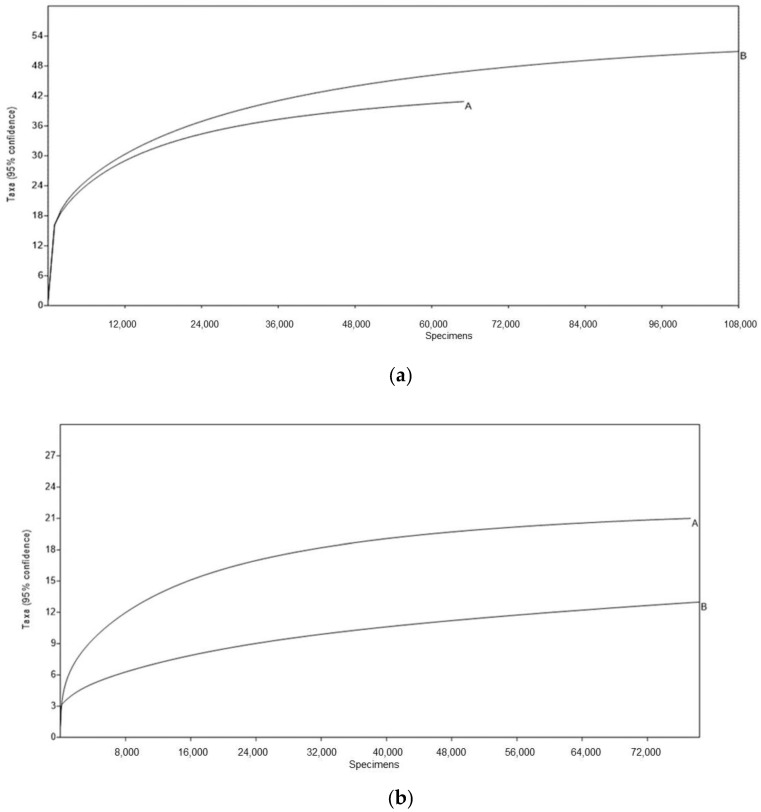
(**a**) Rarefaction curves for the partial sequences for the gene that encodes 16S rDNA of bacteria before incubation (A) and after incubation (B). (**b**) Rarefaction curves for the partial sequences for the ITS 2 region of fungal rRNA before (A) and after (B) incubation.

The rarefaction curves indicated that the overall bacterial and fungal diversity present in both samples was well represented in the obtained results (Figure 7).

Most of the bacterial and fungal OTUs could be assigned to the level of genus based on the homologous sequence alignment method and clustering with sequences obtained from taxonomic databases. 

Ascomycota was the dominant fungal phylum comprising more than 99% of the sequences in both samples, with *Saccharomyces* and *Pichia* being the predominant genera. *Saccharomyces* represented 68% and 56% of the sequences from T1 mini-silos before and after incubation, respectively, while *Pichia* comprised 30% and 44%, respectively. Sequences corresponding to *Aspergillus, Dekkera, Candida, Malassezia, Mortierella, Rhodotorula, Penicillium, Alternaria, Hypocreay*, and *Bionectria* were also found in samples obtained prior to incubation representing approximately 2% of the total sequences. These sequences (genera) were not found in samples obtained from T1 mini-silos after incubation. When comparing *Aspergillus* assigned sequences with sequences corresponding to type strains in GenBank, the closest related sequences (near 99% of similarity) corresponded to species from *Aspergillus* section *Flavi*, which includes *A. flavus*.

**Figure 8:**
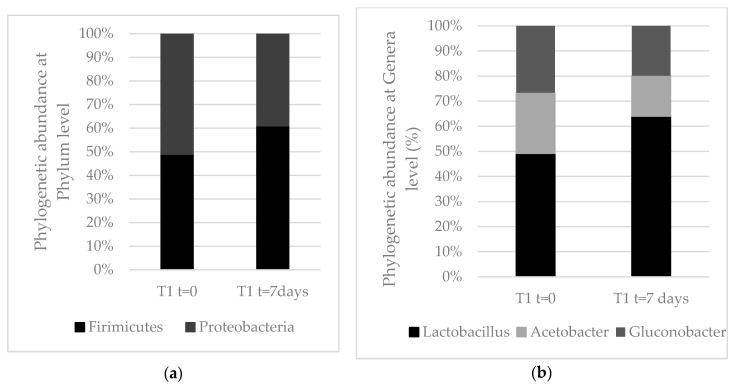
(**a**) Relative abundance of bacteria to Phylum level. (**b**) Relative abundance of bacteria to Genera level.

The taxonomic assignments of the predominant bacterial OTUs from both samples at the level of family and genus are shown in Figure 8. Two bacterial phyla, Firmicutes and Proteobacteria, were dominant (more than 99.9%) in both samples. Less than 0.05% of the sequences obtained before incubation corresponded to other phyla (Deinococcus-Thermus, Actinobacteria, and Cyanobacteria). The abundance of the predominant phyla prior to incubation were very similar (49% for Firmicutes and 51% for Proteobacteria) while after incubation, OTUs in the Firmicutes (61%) were more predominant (Figure 8). 

Alphaproteobacteria, represented by the genera *Acetobacter* and *Gluconobacter*, was the predominant class within Proteobacteria. Less than 0.1% of the sequences corresponded to Beta and Gammaproteobacteria. 

**Figure 9:**
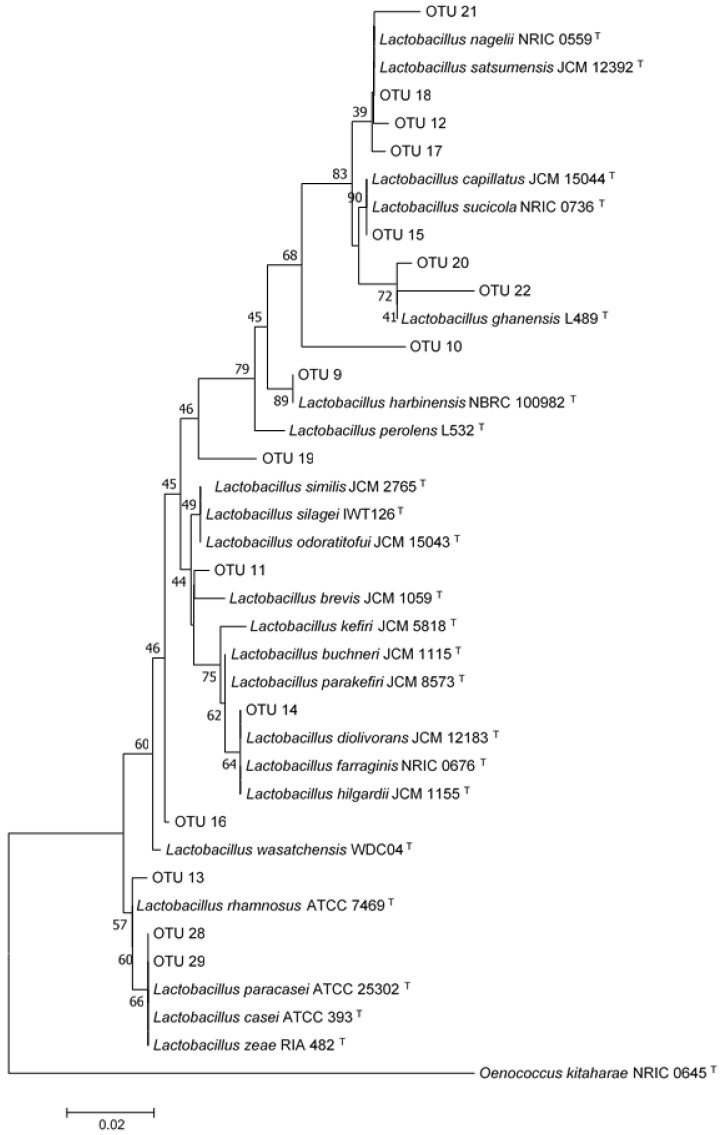
Phylogenetic tree based on OTUs sequences associated with *Lactobacillus* genus. The tree was constructed using the neighbor-joining method. Bootstrap values (1000 tree interactions) are indicated at the nodes. ^T-^ means type strain

The majority of sequences assigned to Firmicutes (more than 99%) belonged to the Lactobacillaceae family. Only 0.003% were assigned to other families (Bacillaceae and Planococcaceae) and were only present in samples obtained before incubation. *Lactobacillus* was the major genus within Firmicutes (more than 99%). Sixteen OTUs corresponding to this genus were detected in both samples. A phylogenetic tree was constructed utilizing the 16 *Lactobacillus* sequences along with sequences of type strains from related species retrieved from GenBank. Some distinct clusters were evident in the phylogenetic tree that were related to the OTUs with sequences corresponding to different species. For example, OTU 18 was affiliated with the *L. nagelii* and *L. satsumensis* group, OTU 9 formed a cluster with *L. harbinensis* type strain, while OTU 28 and OTU 29 were located within the *L. paracasei/L. casei* group (Figure 9). 

**Figure 10:**
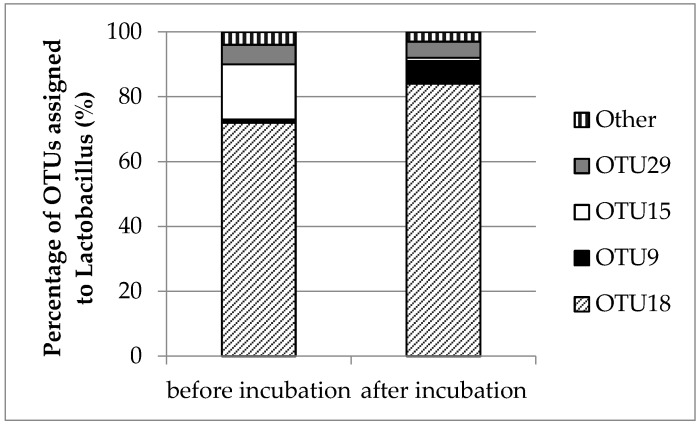
Percentage of sequences assigned to different OTUs in the total of *Lactobacillus* sequences in T1 mini-silos before and after incubation.

The frequency of OTUs assigned to *Lactobacillus* spp. was variable in the two samples. Figure 10 illustrates the percentage of sequences in the samples assigned to different OTUs belonging to *Lactobacillus* sequences. OTU 18 was the predominant sequence in both samples (before and after incubation), but its proportion was higher after incubation. Before incubation OTU 9 represented only 1% of the *Lactobacillus* sequences in that sample, but its proportion increased to 7% after incubation. The proportion of OTU 15 was high (17%) prior to incubation but much lower (1%) after incubation. In contrast, the abundance of OTU 29 was practically the same before and after incubation. 

## 3. Discussion

In this work, the capacity of water kefir to prevent *A. flavus* growth in high-moisture sorghum grain mini-silos during the aerobic phase was demonstrated for the first time. This study also represents the first use of a consortium of organisms rather than a single antagonist as a postharvest biological control preparation, an approach that has been recently emphasized [20]. Antifungal assays were carried out in mini-silos containing ground sorghum grains amended with water, kefir water (WK), or kefir water filtered through a 0.045 µm filter (SWK) and artificially inoculated with a conidial suspension of *A. flavus.* Mini-silos had an open end covered by a sterile 0.45 µm membrane filter to simulate the aerobic phase of the process but not allow infiltration of microorganisms from the surrounding atmosphere. When WK was added to sorghum grains, *A. flavus* biomass after 7 days of incubation at 25 °C was 100 times lower than the level obtained in control mini-silos in which grains were mixed with water. When grains were amended with SWK, which contained no microorganisms, *A. flavus* growth after incubation was similar to the level observed in the control mini-silos. These results indicate that the antifungal activity exhibited in the WK treatment was entirely due to the action of microorganisms present in the kefir water and not to soluble metabolites present in the fermented solution, since no inhibition was observed when sterile kefir water (SWK) was used as a treatment. The enhancement of *A. flavus* growth in mini-silos receiving the SWK treatment suggests that fungal growth in sorghum grains is nutrient limited, since the biomass of *A. flavus* increased when extra nutrients were provided by the sterile kefir water (SWK) treatment. 

Certain bacteria and yeast present in the kefir water (WK) were able to grow on ensiled sorghum grains under the assayed conditions. All the recovered microorganisms proved to be facultative anaerobic organisms that also remained active in the anaerobic phase of the ensiling process. This capacity could be very useful in extending the antifungal action beyond the first aerobic phase to cases where the anaerobic storage is interrupted when a silo bag is opened or breaks [4]. The same bacteria and yeast species were recovered from T1 (WK-treatment) mini-silos before and after incubation. Bacteria were identified as *Lactobacillus* spp., *Acetobacter* spp., and *Gluconobacter* spp. A phylogenetic tree was constructed based on the 16S rRNA sequences of the *Lactobacillus* spp. and type strain sequences from related species. Two of the isolates formed a separate cluster with *Lactobacillus harbinensis* type strain NBRC 100982, suggesting that they could belong to this species. Two other isolates grouped with strains from *Lactobacillus casei* Group and the remaining two isolates formed a separate cluster with *Lactobacillus nagelii* type strain NRIC 0559. The mentioned *Lactobacillus* species could be classified in different groups based on the different pathways they utilize to ferment carbohydrates. *Lactobacillus nagelii* is an obligate homofermentative species, which produces only lactic acid, as a result of the fermentation of hexoses. The other species identified in our study can be classified as a facultative heterofermentative bacterium [32]. These types of bacteria can produce different end products depending on the type of sugars available for fermentation. In the presence of pentoses, they produce lactic and acetic acids but when fermenting hexoses they act as homofermentative bacteria. Homofermentative *Lactobacilli* rapidly decrease pH and increase the concentration of lactic acid in the substrate in which they are growing and have been extensively used as silage inoculants [33]. The antifungal activity of *Lactobacillus* spp. is well documented, and is not only due to the production of organic acids, but also attributed to the production of short chain fatty acids, hydrogen peroxide, reuterin, diacetyl, and bacteriocins [34].

Only two yeast species, *Picha membranifaciens* and *Saccharomyces cerevisiae*, were recovered in culture from T1 mini-silos. Both species have been previously isolated from kefir grains. *Saccharomyces cerevisiae* is commonly used as probiotic [35] while *Pichia membranifaciens* has been selected as biological control agent of fungal diseases in several food matrices. Notably, it has been used as an antagonist against postharvest fungal pathogens during the cold storage of fruits [36,37]. It has been generally considered that yeasts contribute to the aerobic spoilage of silage since some of them can metabolize lactic acid increasing silage pH Duniere et al. (2015) [16], however, demonstrated that inoculation with three *Saccharomyces* strains did not affect aerobic stability of corn silage during the aerobic phase and had no impact on *Lactobacillus* populations or silage quality. Based on these results they proposed that the use of a combination of *Lactobacillus* spp. and *Saccharomyces* strains to produce a new type of inoculant in which yeasts act as probiotic microorganisms to benefit the health and production efficiency of ruminants. The inclusion of yeasts unable to degrade lactic acid in silage could also help to prevent mold growth by consuming oxygen and acting as a biocontrol agent. In this regard, many studies have demonstrated the active role of *Pichia anomala* in preventing *Penicillium roquefortii* growth in airtight stored grain in mini- and medium-scale silos [38].

The results obtained by culture-dependent methods were in accordance with the results obtained by high-throughput sequencing of both bacterial and fungal communities in ensiled sorghum grains amended with kefir water (WK). Firmicutes and Proteobacteria were the predominant bacteria phyla in both analyses. *Lactobacillus* and *Acetobacter* were the most abundant genera within the Firmicutes and Proteobacteria, respectively. This was in agreement with Fiorda et al. (2017) [39] who analyzed and compared the microbial composition of water and milk kefir grains. *Lactobacillus* was the most abundant bacterial genus in both types of grains but *Acetobacter* was mainly found in water kefir, while *Streptococcus* and *Pediococcus* were more abundant in milk kefir.

In the present study, *Lactobacillus* was comprised of 16 different OTUs. Based on the phylogenetic tree, the OTUs could be assigned to different *Lactobacillus* species. The proportion of the predominant OTUs differed prior to and after incubation. OTU 18, which clustered with *L. nagelii* and *L. satsumensis*, was the predominant sequence in both pre- and post-incubation samples, but its proportion was higher after incubation. OTU 9, which most closely matched *L. harbinensis*, also increased its proportion after incubation, while OTU 15, most closely matched to *L. capillatus* and *L. sucicola*, was more abundant prior to incubation. Finally, the proportion of OTU 29, affiliated with the *L. casei/paracasei* group, remained stable. The species matching the most abundant OTUs obtained after incubation, was also isolated in culture before and after incubation. Importantly, species related to OTU 15 and other less abundant OTUs were not present in culture. 

More abundant OTUs corresponded to *Saccharomyces* spp. and *Pichia* spp. which is in agreement with the genera of yeast isolates obtained by culture methods (*S. cerevisiae* and *P. membranifaciens*). Other fungal sequences were also identified in the sequence-based analyses using metabarcoding but they represented a minor proportion of the total number of OTUs. Sequences assigned to *Aspergillus* were not found in samples taken after incubation, which suggests that *A. flavus* biomass represented a very minor proportion of the total fungal biomass in post-incubation samples receiving the WK treatment. This result is not surprising since the yeast concentration in sorghum after incubation increased three-fold, while *A. flavus* biomass was very low, as demonstrated using qPCR.

The results obtained by high-throughput sequencing revealed a more complex microbial community than the obtained by culture methods. Those microorganisms present in low numbers could only be detected by high-throughput sequencing. Their role in biocontrol activity, however, should not be ignored and should be explored in further studies. 

The present work demonstrated, for the first time, that *A. flavus* growth in ensiled sorghum grains can be inhibited by microorganisms from water kefir and that a consortium of microorganisms can be used as a postharvest biocontrol preparation. The water kefir derived microorganisms could grow in mini-silos containing sorghum grains after 7 days of incubation at 25 °C and were mainly represented by three *Lactobacillus* species (*L. harbinensis, L. nagelii*, and *L. casi/paracasei*) and two yeast species (*S. cerevisiae* and *P. membranifaciens*). Based on these results, the use of a mixed inoculum based on the consortium present in kefir water may represent a useful strategy to avoid the growth of *A. flavus* in ensiled sorghum grains, as well as the concomitant contamination with aflatoxins in ensiled sorghum grains. The development of inoculants that combined *Lactobacillus* and yeast has the goal of achieving the benefits of both types of inoculants in one product. In a combined inoculant, lactic or acetic acid produced as a result of *Lactobacillus* spp. primary metabolism would lower the pH of the grains causing inhibition of fungal growth. Moreover, different antifungal compounds produced by *Lactobacillus* spp.—such as phenyllactic acid [40], hydrogen peroxide, proteinaceous compounds [41,42], reuterin [43], fatty acids, and cyclic dipeptides [44]—could also have a role in fungal inhibition. The role of yeast could be related to a rapid oxygen depletion or to the production of soluble and volatile antifungal compounds [13,45,46] and mycocins [47]. In any case, the production of antifungal compounds depends on the matrix and environmental conditions in which microorganisms grow [48], so the presence and the role of such compounds in ensiled grain need to be demonstrated. 

Further studies should be conducted to determine the role of each microorganism in order to generate an inoculant of known and stable composition that could inhibit the growth of undesirable microorganisms and provide additional beneficial effects to the animals fed with sorghum silage.

## 4. Materials and Methods 

### 4.1. Pathogen

A native strain of *Aspergillus flavus* (PJA), belonging to the culture collection from Cátedra de Microbiología, Facultad de Química, UdelaR (Montevideo, Uruguay) was used in this study. The culture was maintained on Potato Dextrose Agar (PDA) at 4 °C.

### 4.2. Quantification of A. flavus Biomass by qPCR

*Aspergillus flavus* biomass was quantified by qPCR in a Rotor-Gene 6000^TM^ (Corbett Life Science, Sydney, Australia) thermocycler according to the protocol described by Shweta et al. (2013) [49] with some modifications. qPCR Reactions were performed in duplicate using a total volume of 10 µL for each sample, consisting of 5µL of Rotor-Gene™ SYBR^®^ Green PCR Master Mix (Quiagen, Venlo, Netherlands), 0.5 µL of primer omt-F (5′-GACCAATACGCCCACACAG-3′) and 0.5 µL primer omt-R (5′-CTTTGGTAGCTGTTTCTCGC-3′) (10 μM each) (2013) [49], 1 µL of template DNA solution and 3 µL sterile miliQ water. The PCR thermal cycling consisted of an initial heating step of 5 min at 95 °C followed by 35 cycles of 95 °C for 30 s for template denaturation and 59 °C for 35 s for primer annealing and extension (during which the fluorescence was measured). After the final amplification cycle, the dissociation curve between 68 °C and 98 °C was obtained to confirm the specificity of the amplification. 

A standard curve was generated by duplicate analysis of five different concentrations of DNA from *A. flavus* PJA. Fungal DNA was extracted using ZR Fungal/Bacterial DNA MiniPrep kit (ZymoResearch, California, CA USA) directly from mycelia obtained from a 48 h-old culture growing on PDA at 28 °C. The double stranded DNA concentration in the obtained solution was determined using Quant-iTdsDNA kit (Invitrogen, California, CA USA) in a Qubit Fluorometer (Invitrogen, California, CA USA). The mean cycle threshold (Ct) values were plotted against the log of the corresponding DNA concentration of each dilution. PCR efficiency was calculated from the slope of the standard curve with the formula efficiency = [10^(−1/slope)^]−1 [50]. Detection limit and dynamic range were set within the linear range. Data analysis was performed using the Rotor-Gene 6000 cycler software version 2.3.1 (Quiagen, Venlo, Netherlands).

### 4.3. Water Kefir Preparation

Water kefir grains (CMUNLP1) were provided by the Cátedra de Microbiología, Facultad de Ciencias Exactas, Universidad de La Plata (La Plata, Argentina). A 4.5% *w*/*v* sugary solution prepared by dissolving unrefined cane sugar (Los Ceibos, Argentina) in distilled water was subsequently inoculated with 10% *w*/*v* kefir grains. The mixture was incubated at 28 °C for 24 h. Kefir grains were then removed by filtration through a plastic sieve to obtain a water kefir (WK) solution. The WK was centrifuged at 3500× *g* for 15 min and the supernatant was subsequently filtered through a 0.45 µm pore size membrane to obtain sterile water kefir (SWK) solution.

### 4.4. Sorghum Grain Preparation and Ensiling

Sorghum mini-silos were prepared as described by Petersson and Schnurer (1995) [51] with some modifications. High tannin sorghum grains (*Sorghum bicolor*) were ground in a blender (Philips Hr2109) at high speed for 30 s, sterilized by autoclaving (121 °C, 15 min) and then dried at 60 °C to a constant weight. Water (control), WK, or SWK was added to the sorghum, in a 1:1 proportion (*w*/*v*) and homogenized with a sterile spatula to obtain the following mixtures: control (sorghum and water), T1 (sorghum and WK) and T2 (sorghum and SWK). The mixtures were dried at 60 °C down to a humidity of 34%. Subsequently, 50 g of each mixture was inoculated with 1 mL of a conidial suspension of *Aspergillus flavus* PJA whose concentration was determined using a hemocytometer. The preparation was then diluted to obtain a final concentration of 5 × 10^5^ conidia/mL. Mini-silos were prepared by compacting the inoculated mixtures in 15 mL sterile centrifuge tubes. A sterile 0.45 µm membrane filter was inserted into the cap of each tube to simulate air leakage. Mini-silos were incubated for 7 days at 25 °C. Additional mini-silos were prepared for the analysis of T1 samples prior to incubation. All treatments were performed in duplicate. 

### 4.5. Analysis of Mini-Silos 

After incubation, mini-silos were analyzed for pH and *A. flavus* biomass. In the case of silos prepared with T1, additional analyses were performed before and after incubation.

The content of each mini- silo was placed in a sterile bag containing 180 mL of sterile water and the mixture was homogenized in a laboratory blender for 1 min (Stomacher 400, Seward, UK). The pH of each homogenate was determined using a pH electrode (HANNA HI1131). Total DNA was extracted from all of the homogenates. One milliliter of each suspension were centrifuged for 1 min at 10,000 rpm. Pellets were reconstituted in 750 µL of a lysis solution and total DNA was extracted from the pellets using the ZR Fungal/Bacterial DNA MiniPrep kit (ZymoResearch). Aliquots of DNA solutions obtained from the samples were used to determine the concentration of *A. flavus* by qPCR, as described above. All determinations were performed in duplicate. Additional aliquots of total DNA solutions obtained from T1 mini- (initial time and after 7 days of incubation) were used to characterize the microbial community using a metabarcoding approach, as described below. The concentration of yeast and lactic and acetic acid bacteria in mini-silos prepared with T1 were determined before and after the incubation period. Microbiological analyses were performed by plate counting on Potato Dextrose Agar (Oxoid Ltd., England) amended with 0.017% of chloramphenicol (Sigma-Aldrich, Missouri, MO USA) and Man, Rogosa, and Sharpe Agar (Oxoid Ltd., Hampshire, England) with 0.04% cycloheximide (Sigma-Aldrich, Missouri, USA), respectively. Plates were incubated for four days at 28 °C, in aerobic and anaerobic conditions. Every bacterial or yeast colony that exhibited a different morphology was isolated using either aerobic or anaerobic conditions and identified to species level as described below.

Results were analyzed by ANOVA and significant differences between means were determined by an LSD test at a significance level of 0.05 using the INFOSTAT software package version 2017 (https://www.infostat.com.ar) (Grupo InfoStat, FCA, Universidad Nacional de Córdoba, Argentina, 2009). 

### 4.6. Molecular Identification of Yeast

Yeast isolates were identified by sequence analysis of the D1/D2 variable domain. DNA extraction was carried out as described by Schena et al. (1999) [52]. PCR fragments were generated using primers ITS1 [53] and D2R [54] as described by Martinez et al. (2016) [55]. PCR products were visualized by electrophoresis on 0.8% agarose gel. Nucleotide sequences of the PCR products were obtained with primer D2R at Macrogen (Macrogen Inc., Seoul, Korea) and compared to NCBI databases using BLAST (http://www.ncbi.nlm.nih.gov/).

### 4.7. Molecular Identification of Bacteria

Lactic and acetic acid bacteria were identified by sequence analysis of a partial sequence of 16S ribosomal DNA. Alkaline lysis was carried out for DNA extraction. Bacterial cultures grown in Man, Rogosa, and Sharpe Agar (MRS, Oxoid Ltd., England) for 48 h were suspended in 100 µL of a NaOH solution (0.05 M) and incubated for 15 min at 95 °C. The suspensions were then centrifuged at 1000 rpm for 2 min and the supernatants were retained. PCR reactions were performed in a MultiGene Mini Personal TC020-24 thermocycler (Labnet International Inc., New Jersey, NJ USA) in a total reaction volume of 25 µL consisting of 2.5 µL of 10× buffer, 2.5 µL of dNTPs (2 mM each), 1.5 µL of MgCl_2_ (25 mM), 1 µL of primer 27F (10 µM) [56], 1 µL of primer 1492R (10 µM) [56], 0.2 µL of Taq DNA Polymerase (2.5 U/µL) (Thermo Fisher Scientific, Massachusetts, MA USA), 1 µL of bacterial lysis supernatant and sterile MiliQ water up to 25 µL. The PCR protocol consisted of an initial heating step of 5 min at 94 °C, followed by 35 cycles of 1 min at 94 °C for template denaturation, 2 min at 55 °C for primer annealing, and 3 min at 72 °C for primer extension, with a final extension at 72 °C for 10 min. PCR products were visualized by electrophoresis on 0.8% agarose gel. Amplicons were purified and sequenced in forward direction at Macrogen (Macrogen Inc., Seoul, Korea). Nucleotide sequences of the PCR products were compared to NCBI databases using BLAST (http://www.ncbi.nlm.nih.gov/).

### 4.8. Phylogenetic Analysis 

Phylogenetic analysis of D1/D2 sequences for yeast and ribosomal RNA 16S sequences for bacteria were conducted using MEGA version 7 (University, Pennsylvania, PA USA). DNA sequences were aligned with sequences of homologous regions of closely related type strains retrieved from GenBank. In all cases, evolutionary distances were computed using Jukes–Cantor method, and phylogenetic trees were obtained by neighbor-joining. Stability of clades was assessed with 1000 bootstrap replications. 

### 4.9. Characterization of the Fungal and Bacterial Microbiota Using Metabarcoding and High-Throughput Sequencing 

Characterization of the microbiota of T1 mini-silos before and after incubation were conducting by metabarcoding and high-throughput sequencing. Genomic DNA was extracted directly from the product using the same extraction kit as described above, following the manufacturer’s instructions. Total DNA from each sample was quantified in a spectrophotometer (λ = 260 nm) (Nanodrop; Thermo Fisher Scientific Inc.) and the DNA concentration was adjusted to 5.0 ng/µL. The ITS 2 region of fungal rRNA was amplified with the universal primers ITS3/KYO2 and ITS4 [57]. The V4 region of bacterial 16S rRNA was amplified using the universal primers 515F and 806R [58]. A pair of peptide-nucleic-acids (PNA) were incorporated into the PCR amplification to reduce the generation of non-target chloroplast and mitochondrial amplicons. All primers were modified to include Illumina adaptors (www.illumina.com). PCR reactions were conducted in a total volume of 25 µL containing 12.5 μL of KAPA HiFi HotStart ReadyMix (Kapa Biosystems, Wilmington, MA, USA), 1.0 μL of each primer (10 μM), 2.5 μL of DNA template, and 8.0 μL nuclease free water. Reactions were incubated in a T100 thermal cycler (Bio-Rad, California, CA USA) for 3 min at 98 °C followed by 30 cycles of 30 s at 95 °C, 30 s at 50 °C, and 30 s at 72 °C. All reactions ended with a final extension of 1 min at 72 °C. Nuclease-free water (QIAGEN, Valencia, CA, USA) replaced template DNA in negative controls. All amplicons and amplification mixtures including negative controls were sequenced on a MiSeq platform using V2 chemistry (Illumina, San Diego, CA USA). 

### 4.10. Data Analysis of Amplicon Sequences

Paired-end reads were merged using PEAR 0.9.6 paired-end read merger [59] and default parameters. The CLC genomics workbench V8 (Qiagen) was used for primer and quality trimming with a minimum of Q20. Sequences without either primer were discarded. Chimeric sequences were identified and filtered using VSEARCH 1.4.0 [60]. The UCLUST algorithm [61] of the software package QIIME 1.9.1 [62] was used to cluster sequences at a similarity threshold of 97% against the UNITE dynamic database released on 31.01.2016 [63] for ITS2 reads and Greengenes Database for the 16S rRNA reads. Singletons were removed and sequences that failed to cluster against the database were de novo clustered using the same algorithm. The most abundant sequences in each OTU were selected as representative sequences and used for the taxonomic assignment using the BLAST algorithm [64] as implemented in QIIME 1.9.1. The OTU table was normalized by rarefaction to an even sequencing depth to remove sample heterogeneity. The rarefied OTU table was used to calculate alpha diversity indices, including Chao1 and Shannon metrics, using the PAST program [65]. A phylogenetic tree was generated for the OTUs corresponding to *Lactobacillus* genera using MEGA version 7, including sequences from type strains retrieved from GenBank. The neighbor-joining method was used and evolutionary distances were computed using Jukes–Cantor method. Stability of clades was assessed with 1000 bootstrap replications. 

## Figures and Tables

**Table 1:** Alpha diversity indices (Chao1 and Shannon) for bacterial and fungal communities, in T1(water kefir) mini-silos, before (A) and after (B) incubation

	Sample	Sequences	OTUs	Chao1	Shannon
Bacteria	A	66571	41	42.7	1.681
	B	109365	51	52.5	1.613
Fungi	A	77432	21	21.2	0.1383
	B	78539	13	16.0	0.2657
